# Association of Left Atrial Sphericity with Risk of Stroke in Patients with Atrial Fibrillation. Sub-Analysis of the ASSAM Study

**DOI:** 10.1007/s13239-021-00587-y

**Published:** 2021-11-08

**Authors:** Katarzyna Dudzińska-Szczerba, Marta Zalewska, Wojciech Niemiro, Ilona Michałowska, Roman Piotrowski, Agnieszka Sikorska, Piotr Kułakowski, Jakub Baran

**Affiliations:** 1 Department of Cardiology, Centre of Postgraduate Medical Education, Grochowski Hospital, Warsaw, Poland; 2 Department of Environmental Hazards Prevention and Allergology, Medical University of Warsaw, Warsaw, Poland; 3 Faculty of Mathematics, Informatics and Mechanics, University of Warsaw, Warsaw, Poland; 4 Department of Radiology, Institute of Cardiology, Warsaw, Poland; 5 Division of Clinical Electrophysiology, Department of Cardiology, Centre of Postgraduate Medical Education, Grochowski Hospital, Grenadierów Street 51/59, 04-073 Warsaw, Poland

**Keywords:** Left atrium, Sphericity, Atrial fibrillation, Ischemic stroke, Computed tomography

## Abstract

**Purpose:**

The study was designed to evaluate the value of left atrial (LA) sphericity (LASP) in the identification of patients with atrial fibrillation (AF) who had prior ischemic stroke. The secondary aim was to investigate the possibility of improving stroke risk assessment based on six geometrical variables of LA.

**Methods:**

This prospective observational study involved 157 patients: 74 in the stroke group and 83 in the control. All patients had cardiac computed tomography (CT) performed to analyze LA volume and dimensions. LASP and the discriminant function of six geometrical measurements were calculated.

**Results:**

Multivariate logistic regression analysis showed a significant association of stroke with and gender, diabetes, CHA2DS2-VASc score, LA anteroposterior diameter, and LA sphericity. Patients with prior stroke had lower LASP than those without (66.6 ± 10.3% vs. 70.5 ± 7%; *p* = 0.0062). The most accurate identification of patients with a history of ischemic stroke was achieved by using a function of six geometrical measurements, the sphericity and volume coefficient. The C-statistic was higher for the above discriminant function (0.7273) than for LASP (0.3974). The addition of the discriminant function to the CHA2DS2-VASc score increased the performance of the risk score alone.

**Conclusion:**

LASP is associated with prior stroke in AF patients. The proposed new formula for identification of AF patients who are at risk of stroke, based on geometrical measurements of LA, is superior to the basic LASP in identification of AF patients with a history of stroke.

**Supplementary Information:**

The online version contains supplementary material available at 10.1007/s13239-021-00587-y.

## Introduction

Atrial fibrillation (AF) is the most common arrhythmia, and has become an increasingly serious public health problem in an aging population. It doubles the risk of death and increases the risk of ischemic stroke fivefold.^7, 14^ Stroke is the main cause of long-term disability, and is a serious problem for healthcare systems.^9^ It has been estimated that 20% of ischemic strokes are caused by cardiac embolisms, most often in the course of AF.^6^

It is common practice to assess the risk of thromboembolic (TE) events in patients with AF by calculating the CHA_2_DS_2_-VASc score. However, multiple studies have shown that the score allows only moderately for the identification of patients at risk of stroke or peripheral embolism.^5, 13^ Thus, new factors are still being searched for to improve stratification of the risk of TE in the AF population.

The left atrial sphericity (LASP) is a novel parameter based on the analysis of the shape of the left atrium (LA) which depicts the difference between real LA geometry and a perfect sphere. The LA structural remodeling results in chamber dilation, alterations in the cardiomyocyte, fibroblast, and noncollagen infiltrative components of the atrium, but also changes in LA shape.^3, 11^ The LASP has been shown to be an independent predictor of arrhythmia recurrence after AF ablation. It may be also important in the identification of patients with an increased risk of TE, however, the data on this subject are limited.^3, 4, 11^

The primary aim of our study was to evaluate the value of LASP in identification of patients with AF who had prior ischemic stroke. The studied patient population was well-defined, with a known anticoagulation regimen at the time of neurological event. The secondary aim was to investigate the possibility of improving stroke risk assessment based on 6 geometrical variables derived from computed tomography (CT) of LA, using different statistical methods.

## Methods

### Patients and Study Design

**Figure 1:**
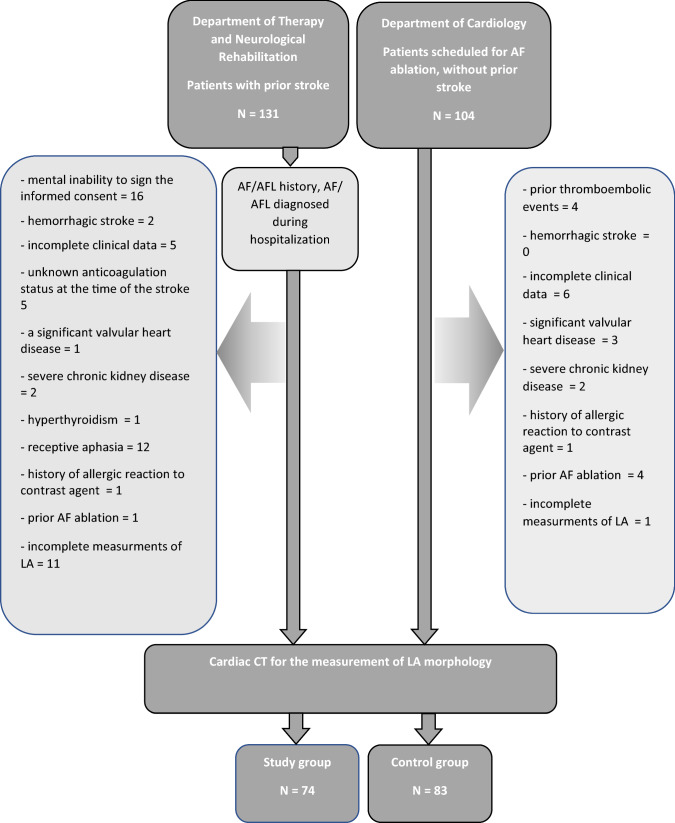
A schematic description of study design. AF, atrial fibrillation.

The study design was previously described.^2^ Briefly, this prospective observational study included 74 consecutive randomly chosen patients after ischemic stroke, with AF, who were hospitalized in the neurological rehabilitation department after experiencing ischemic stroke between 2014 and 2016. The control group consisted of 83 randomly chosen subjects with AF, without a history of TE, scheduled for AF ablation in the cardiological ward (Fig. 1). In addition to the previously described exclusion criteria, patients with incomplete measurements of LA were also excluded from the present analysis. The study was approved by the local Ethical Committee of Centre of Postgraduate Medical Education and registered in the National Clinical Trial database (NCT02654795). Written informed consent was obtained from all patients.

### Imaging Protocol

CT angiography was performed with a dual-source CT scanner (Somatom Definition Flash; Siemens Medical Solutions, Erlangen, Germany), using prospective ECG gating. High-concentration iodinated contrast material (≥ 350 mg/mL) was administered into the antecubital vein in the amount of 70–80 mL, using a power injector, at a flow rate of 5 mL/s, followed by a 30-mL saline chaser. Image acquisition was initiated 5 s after reaching 100 HU threshold enhancement within the region of interest placed in the left atrium. Prospective ECG-gated examinations were centered at the best diastolic phase of the R-R interval. Patients did not receive β-blockers to control heart rate.

### Image Analysis

**Figure 2:**
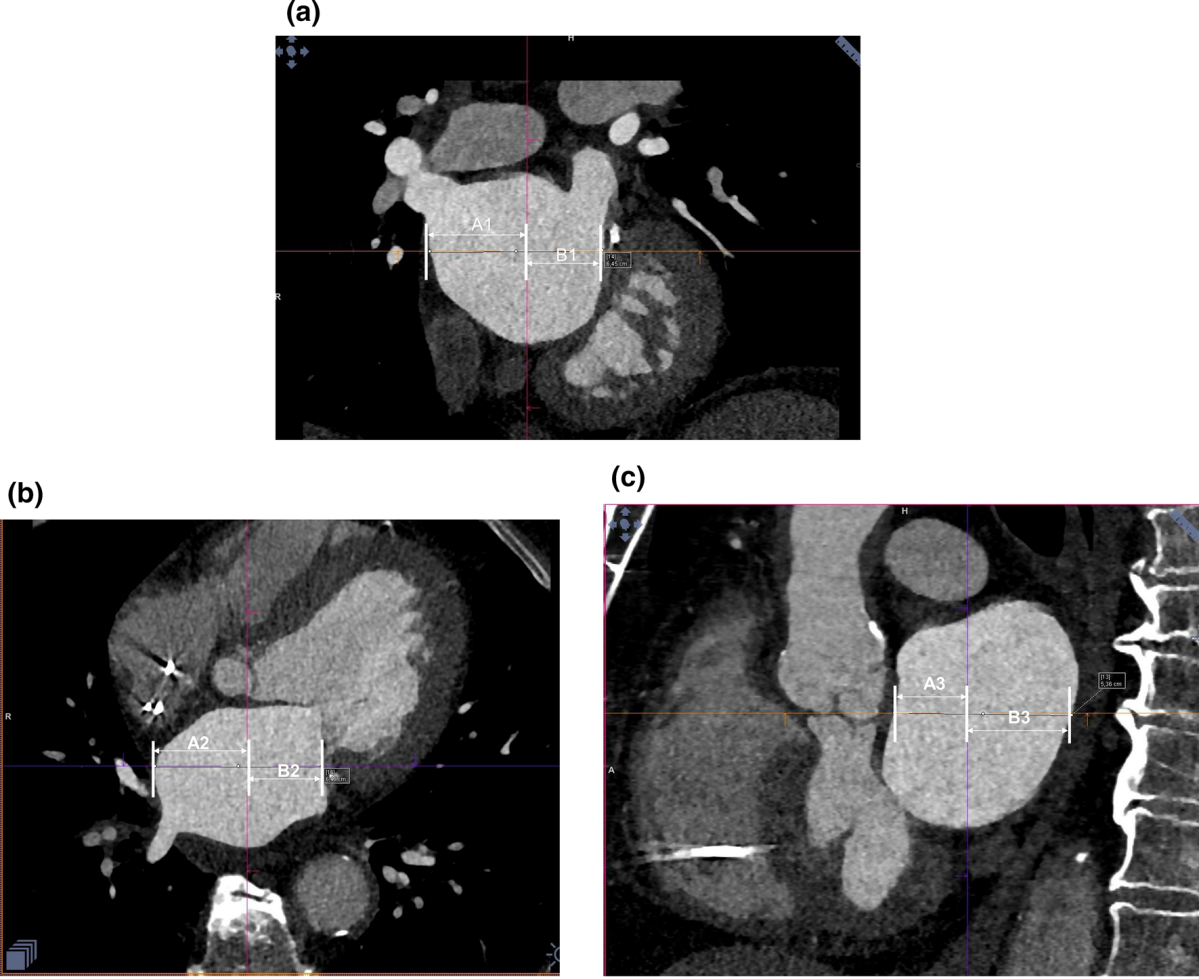
Three planes of left atrium (LA) on computed tomography, based on the principal axis (LA transverse diameter, LA superior-inferior dimension and LA anteroposterior diameter), and the center of mass; A1 + B1 = LA transverse diameter (a); A2 + B2 = LA superior-inferior dimension (b); and A3 + B3 = LA anteroposterior diameter (c) divided by the center of mass of LA.

The analysis of anatomy and measurements of LA size were performed on 1-mm slices, using commercially available software (Leonardo, Siemens Medical Systems). The LA was compared to a fitted sphere according to Bisbal *et al.*^4^ The radius of the sphere which best fit the LA shape was calculated as the mean of distances between points of the LA wall in three planes, based on the principal axis (LA transverse diameter, LA superior-inferior dimension and LA anteroposterior diameter), and the center of mass (average radius [AR], AR = mean (*A*_1_, *B*_1_, *A*_2_, *B*_2_, *A*_3_, *B*_3_), where *A*_1_ + *B*_1_ = LA transverse diameter; *A*_2_ + *B*_2_ = LA superior-inferior dimension; and *A*_*3*_ + *B*_3_ = LA anteroposterior diameter divided by the center of mass of LA) (Figs. 2a–2c). In accordance with the formula proposed by Bisbal *et al.*,^4^ the coefficient of variation of the sphere (CVS = SDR/AR, where SDR = sd (*A*_1_, *B*_1_, *A*_2_, *B*_2_, *A*_3_, *B*_3_), sd—standard deviation) was obtained to define the LASP *via* the formula LASP =  (1 − CVS) × 100. The interpretation of volume coefficient (VC) as an estimate of the volume of LA is explained by the fact that (2 × *π*/9) × VC is the mean of the volumes of spheres with the radii *A*1, *B*1, *A*2, *B*2, *A*3, *B*3.

The discriminant function calculated by the R function lda is the following: 0.0161 × *A*1 − 0.0192 × *A*2 + 0.1076 × *A*3 − 0.0016 × *B*1 − 0.0694 × *B*2 + 0.1321 × *B*3 − 0.1102 × LASP + 0.0061 × VC/10000

### Statistical Analysis

Data are presented as frequencies and percentages for categorical variables, and as means ± standard deviations (for approximately normally distributed variables), or medians and inter-quartile ranges (25–75th), (skewed distribution) for continuous variables. The *χ*^2^ or Fisher exact tests were used to compare proportions between the groups. Student's t-test and Welch's t-test were used to compare continuous variables between groups according to normality assumptions. Collinearity between variables was assessed using the Pearson correlation coefficient. Univariate analysis and multivariate logistic regression analysis were performed to identify factors predictive of stroke. Finally, receiver operator characteristic curves were constructed, and *c*-statistics (area under curve) were calculated to evaluate the value of specific factors for identification of patients with a history of ischemic stroke. A *p* value of ≤ 0.05 was considered significant. All analyses were conducted using R language and environment for statistical computing.^12^ For the discriminant analysis, the lda function available in the R package (library MASS) was used. The lda function enables a calculation of the probability of belonging either group, using the method of cross-validation.

## Results

The study involved 157 patients: 74 in the stroke group (71.4 ± 11.0 years) and 83 in the control group (61.4 ± 10.1 years). Table 1 shows baseline patients characteristics.

**Table 1:** Baseline patient characteristics.

	Control group *N* = 83	Stroke group *N* = 74	*p*
Age (years)	61.4 ± 10.1	71.4 ± 11.0	< 0.0001
Male (%)	69.9	55.4	0.0873
Hypertension (%)	44.6	87.8	< 0.0001
Diabetes (%)	5.95	24.69	0.0017
Congestive heart failure (%)	8.4	41.9	< 0.0001
LA transverse diameter (mm)	72.4 ± 6.4	77.04 ± 10.4	0.0086
LA superior-inferior dimension (mm)	60.11±6.37	59.38±10.41	0.6023
LA anteroposterior diameter	42.72±7.23	47.47±11.03	0.0020
Volume of LAA (cm^3^)	10.02±4.52	12.2±7.57	0.0339
LA sphericity	70.54 ± 6.95	66.58±10.34	0.0062
Volume of LA (cm^3^)	124.12 ± 37.56	149.37 ± 95.45	0.0356
Morphology of LAA			
1	27 (32.5%)	29 (39.1%)	0.0636
2	24 (28.9%)	9 (12.2%)	
3	14 (16.9%)	10 (13.5%)	
4	18 (21.7%)	23 (31.1%)	
HAS BLED	1.6 ± 1.3 1.0 [0.0–1.0]	3.0 ± 1.0 3.0 [2.0–4.0]	< 0.0001
CHA_2_DS_2_-VASc score	1.60 ± 1.29 1.0 [1.0–2.0]	3.7 ± 1.7 4.0 [2.0–5.0]	< 0.0001
CHA_2_DS_2_-VASc score = 0 (%)	43.4	4.0	
CHA_2_DS_2_-VASc score ≤ 1 (%)	24.1	12.2	
CHA_2_DS_2_-VASc score > 2 (%)	32.5	83.8	< 0.0001
Medications			
RAAS inhibitors (%)	49 (59.04)	46 (62.16)	0.8131
OAC (%)	76 (91.57)	23 (31.08)	< 0.0001
Proper anticoagulation therapy^a^	72 (86.75)	10 (13.51)	< 0.0001
Antiarrhythmic drugs (%)	49 (59.04)	4 (5.41)	< 0.0001
β-Blocker (%)	55 (66.27)	39 (52.7)	0.0989

LA, left atrial; LAA, left atrial appendage; the LAA morphology was divided into four types: 1 – chicken wing; 2 – windsock. 3 – cauliflower; 4 – cactus; RAAS, Renin-angiotensin-aldosterone system; OAC, oral anticoagulant

^a^Novel oral anticoagulants at standard dose or vitamin K antagonist with therapeutic INR levels at admission

Compared with the control group, the patients in the stroke group were older (*p* < 0.0001), had a higher CHA_2_DS_2_-VASc score (3.7 ± 1.7 vs. 1.60 ± 1.29, *p* < 0.0001) and HAS-BLED score (risk of bleeding complications) (3.0 ± 1.0 vs. 1.6 ± 1.3, *p* < 0.0001). Furthermore, the patients in the stroke group were more prone to comorbidities such as hypertension, congestive heart failure, and diabetes mellitus (*p* < 0.0001 for all). The patients in the stroke group had a greater LAA volume (12.2 ±7.6 vs. 10.0 ± 4.5 cm^3^, *p* = 0.0339) and LA (149.4 ± 95.5 vs. 124.1 ± 37.6 cm^3^; *p* = 0.0356). They also had a greater LA transverse diameter (77.0 ± 10.4 vs. 72.4 ± 6.4 mm; *p* = 0.0086) and LA anteroposterior diameter (9.2 ± 3.8 vs. 7.2 ± 2.9 mm; *p* = 0.002).

The mean heart rate during CT imaging was 73.80 in the stroke group and 64.99 in the control group (*p* value = 0.0117). In the 59 patients (80.82%) within the study group and 71 (91.03%) in the control group, CT scans were obtained during sinus rhythm (*p* value = 0.01174), in the remaining patients AF was recorded. (There are no data for 5 patients in the control group, and 1 patient in the study group).

In the univariate analysis, parameters significantly associated with previous stroke included diabetes mellitus, hypertension and congestive heart failure, CHA_2_DS_2_-VASc score, volume of LA and LAA, LA transverse and anteroposterior diameter as well as LA sphericity (Table 2).

**Table 2:** Univariate analysis of risk factors of stroke.

	OR	95% CI	*p* value
Male	0.54	0.26–1.09	0.0698
Diabetes	54.76	8.51–2279.71	< 0.0001
Hypertension	8.84	3.75–22.95	< 0.0001
Congestive heart failure	7.72	3.01–22.59	< 0.0001
CHA_2_DS_2_-VASc score	17.83	5.18–95.69	< 0.0001
Volume of LA	1.01	1.00–1.02	0.029
LA transverse diameter	1.04	1.01–1.08	0.0099
LA superior-inferior dimension	0.99	0.95–1.03	0.591
LA anteroposterior diameter	1.06	1.02–1.10	0.0027
Volume of LAA	1.06	1.00–1.13	0.0375
LAA morphology (non-CW)	0.70	0.34–1.42	0.6999
LA sphericity	0.94	0.91–0.99	0.0066

LA, left atrial; LAA, left atrial appendage; CW, chicken wing

Multivariate logistic regression analysis including demographic, clinical variables and LA and LAA morphological parameters, revealed that the variables associated with stroke were gender (OR 6.50; [1.03–41.03]; *p* = 0.0421), diabetes (OR 72.01; [4.77–1086.97]; *p* = 0.016), CHA2DS2-VASc score (OR 4.67; [2.09–10.40]; *p* = 0.0001), LA anteroposterior diameter (OR 1.24; [1.06–1.44]; *p* = 0.0050) and LA sphericity (OR 0.76; [0.66–0.87]; *p* = 0.0001) (Table 3).

**Table 3:** Multivariate logistic regression analysis to predict stroke.

	OR	95% CI	*p* value
Male	6.50	1.03–41.03	**0.0421**
Diabetes	72.01	4.77–1086.97	**0.0016**
Hypertension	2.44	0.43–13.80	0.3031
Congestive heart failure	0.41	0.06–2.87	0.3612
CHA_2_DS_2_-VASc score	4.67	2.09–10.40	**0.0001**
Volume of LA	1.01	0.98–1.04	0.5044
LA transverse diameter	0.92	0.83–1.01	0.0765
LA superior-inferior dimension	1.02	0.93–1.13	0.6551
LA anteroposterior diameter	1.24	1.06–1.44	**0.0050**
Volume of LAA	1.01	0.89–1.14	0.9290
LAA morphology (non-CW)	0.95	0.55–1.66	0.8561
LA sphericity	0.76	0.66–0.87	**0.0001**

Significance of bold value is *p* < 0.05

LA, left atrial, LAA, left atrial appendage; CW, chicken wing

**Figure 3:**
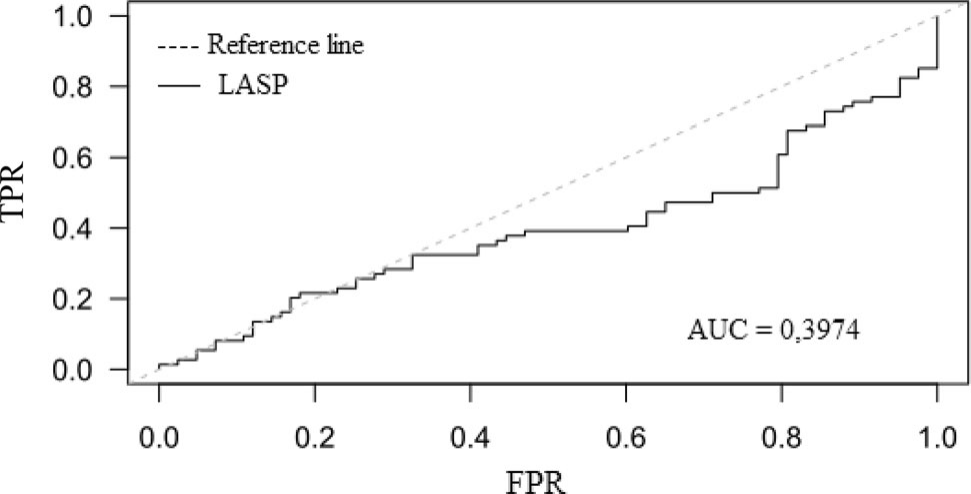
ROC curves for predicting stroke. ROC curve analysis showing the prognostic value of LASP (FPR, False-Positive Rate, TPR, True-Positive Rate, AUC, area under the curve, LASP, left atrial sphericity).

**Figure 4:**
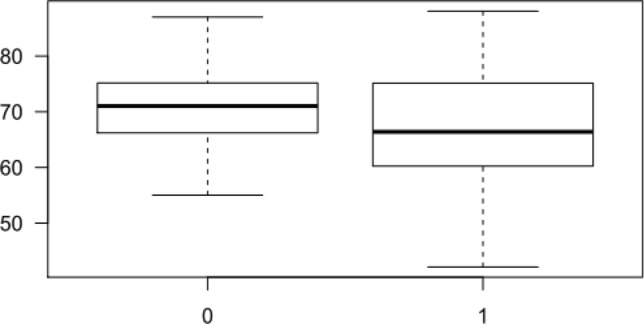
Boxplots of LASP for patients with stroke (1) and control (0).

Patients with prior stroke had lower LASP than those without (66.6 ± 10.3 vs. 70.5 ± 6.9%; *p* = 0.0062). The ROC curve, and corresponding area under the ROC curve (AUC) for the LASP, showed that higher values of the LASP correspond to a smaller proportion of patients with previous stroke (almost for the entire curve False-Positive Rate > True-Positive Rate, AUC 0.3974, 95% CI 0.3062–0.4887) (Fig. 3). The same trends can be seen in Fig. 4.

**Figure 5:**
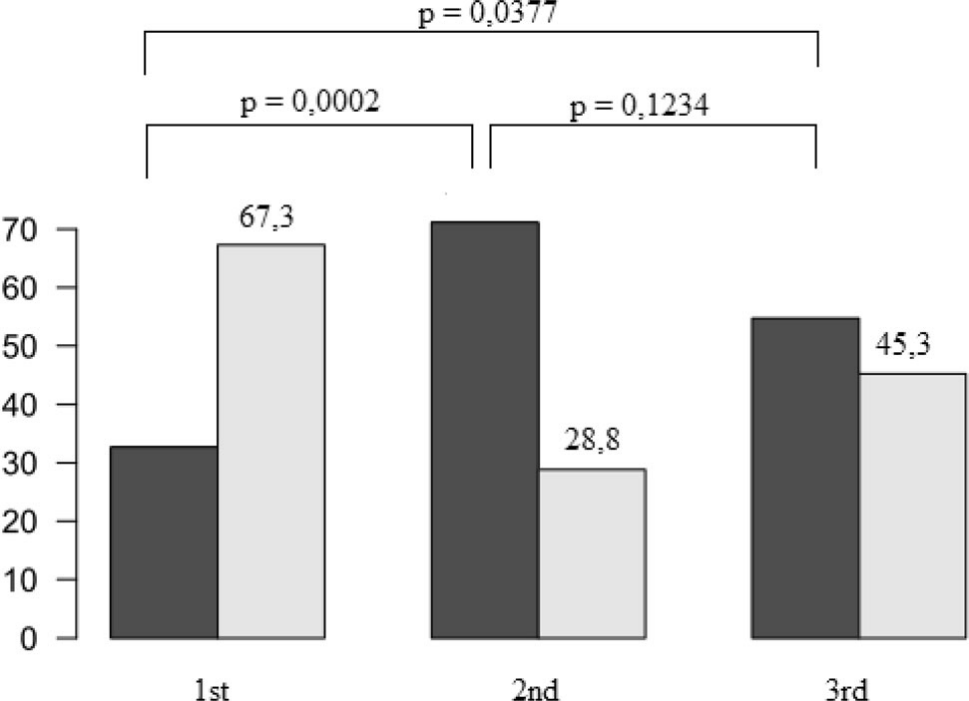
Percent of patients in the stroke group (light grey) and control (dark grey) in the three tertiles of LASP.

Patients with lower LASP had significantly more often previous TE compared to those with higher values (67.3 vs. 28.8 vs. 45.3% for the first, second and third tertile of LASP, respectively, *p* = 0.0377) (Fig. 5).

After limiting the analysis to geometrical variables derived from CT of LA the most accurate identification of patients with a history of ischemic stroke was achieved by the use of a function of six geometrical measurements of the LA (A_1_, B_1_, A_2_, B_2_, A_3_, B_3)_, the sphericity coefficient and volume coefficient.

**Figure 6:**
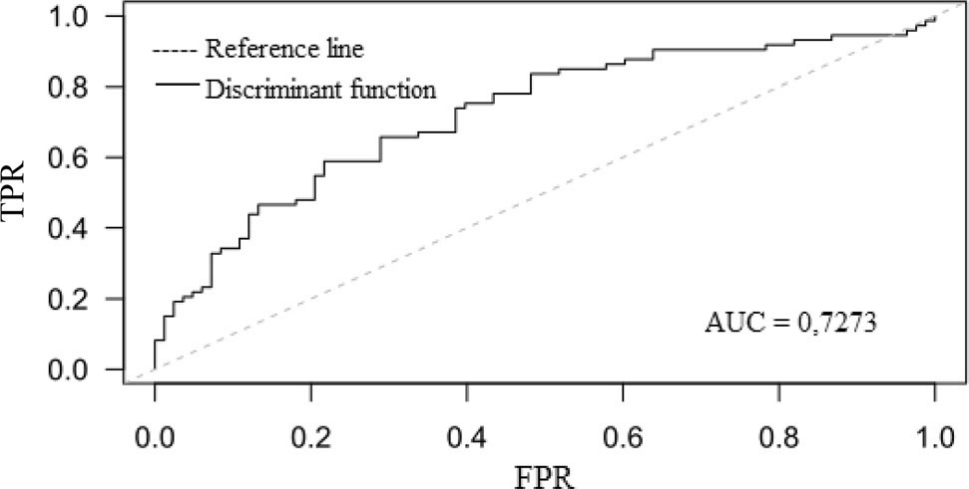
ROC curve based on the estimated probabilities of belonging to the stroke group for discriminant function (FPR, False Positive Rate, TPR, True-Positive Rate, AUC, area under the curve).

In ROC analysis, the AUC was 0.7273 (95% CI 0.6475–0.8071) for the above discriminant function for identification of patients with previous stroke. The cut-off value of (− 0.0863) had a sensitivity of 77% and a specificity of 65% in identifying patients with previous stroke (Fig. 6).

**Figure 7:**
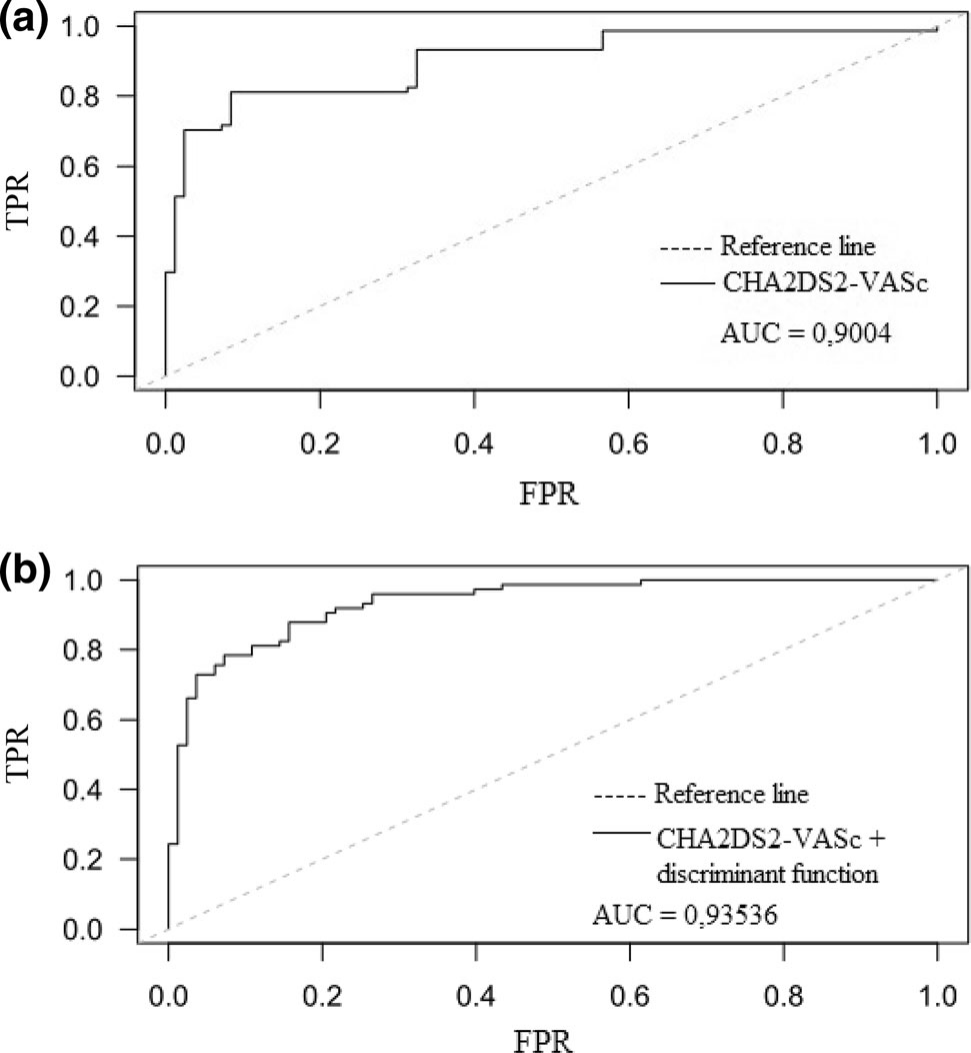
ROC curves for the prediction of stroke for CHA_2_DS_2_-VASc score (a) and after addition of the discriminant function to the CHA_2_DS_2_-VASc score (b).

The addition of the discriminant function to the CHA_2_DS_2_-VASc score (calculated before stroke onset) increased the performance of the risk score alone: the AUC was 0.9004 and 0.9357, respectively (Fig. 7).

Patients receiving proper anticoagulation therapy as in-home medication (received novel oral anticoagulants at a standard dose, or a vitamin K antagonist while maintaining therapeutic INR levels on admission) before stroke onset have been additionally analyzed and compared with others in the stroke group. Anticoagulation had no impact on the results (Fig. 1S in Supplement Data).

## Discussion

In this study, the association between the LA shape and the risk of stroke was found using a simple computation of the anatomical measurement of the LA based on CT imaging. The study showed the addition of LASP to CHA_2_DS_2_-VASc score might further improve identification of patients at risk of TE. Moreover, it was found that a function of six geometrical measurements, the sphericity coefficient and volume coefficient, had a greater ability than LASP alone to identify patients at risk of TE. The clinical use of this parameter may be particularly valuable in patients classified as low risk based on the CHA_2_DS_2_-VASc score system, in whom antithrombotic therapy is not recommended. The inclusion of the LA shape may lead to reclassification of risk, and therapy indication.

AF is an important factor associated with LA dilatation, and the association of LA enlargement and AF has been well recognized. The standard for linear LA measurement is the M-mode or 2-D derived anteroposterior linear dimension.^8^ However, this measurement may not reflect the volume of LA. The measurement of LA volume is currently recommended to assess the size of LA and remodeling. LA volume reflects long-term LA exposure to elevated left ventricular filling pressure. Therefore, the size of LA, especially expressed as its volume, is a valuable parameter enabling long-term hemodynamic monitoring of patients and is a strong predictor of outcomes.^8^ Although echocardiography is the most widely used method for evaluation of the LA, CT is emerging as a valuable method for assessing the shape and size of LA. 2D echocardiography significantly underestimates LA volumes, in comparison with cardiac CT.^1^ Previous studies have shown that some anatomical and functional LA and LAA features are independently associated with prevalent stroke in a population of patients with AF. The results of studies examining the relationship between the LASP and stroke are conflicting. In a recent study, three-dimensional LA shape assessed by means of LASP was identified as an independent predictor of recurrence after AF ablation and increased risk of TE.^3, 4, 11^

Nunes *et al.* showed that more spherical LA shape was independently associated with an increased risk for TE in a specific population with rheumatic mitral stenosis.^10^

Our findings differ from those reported by Bisbal *et al.*^3^ This discrepancy may be due to differences in statistical approach used in our study. We calculated the proportion of strokes in the three tertiles of LASP. Patients with lower LASP had significantly higher proportion of previous TE compared to those with higher values. We conducted a Wilcoxon-Mann-Whitney test to verify the hypothesis about the lack of differences between the stroke and control groups. The test rejects this hypothesis and favors the alternative hypothesis, saying that the LASP in the stroke group is lower (*p* = 0.0239). This confirms our previous findings that higher LASP is associated with a lower probability of stroke. Another reason for different results obtained by our study and Bisbal *et al.* is the fact that we studied a slightly older patient population. Furthermore, Bisbal *et al.* study was based on smaller sample size (only 29 patients in the study and control groups) and this was a highly selected, low-risk AF population (the study population presented a high proportion of low (CHA_2_DS_2_-VASc ≤ 1) or very low (CHA_2_DS_2_-VASc = 0) anticipated stroke risk (82.8 and 43.1%, respectively). The authors pointed out that LA imaging was not conducted at the time of TE, the sphericity and volume of LA change over time.^4^ In our study, CT imaging in the vast majority of patients in the study group was performed within one month of the onset of stroke during hospitalization in the neurorehabilitation ward.

Our study showed that presenting data in the form of six geometric measurements of the LA allowed for identification of patients with prior stroke with the probability of correct diagnosis of 70%. We presented a discriminant function of six geometrical measurements, the sphericity coefficient and volume coefficient which provided the most satisfactory quality of identification of patients with previous stroke, and had a better accuracy compared to the LASP. The probability of incorrect classification (PIC) using this function and for the optimal selection of the threshold was 0.3185. Because this result was obtained using the cross-validation (leave-one-out) method, this allowed us to conclude that 0.3185 is an approximately unbiased error estimate for stroke risk assessment for a new patient.

There are several potential limitations of our study. Firstly, although the study prospectively recruited patients with a history of ischemic stroke, data concerning their medication at the time of stroke has been derived from the patient files which might have influenced the accuracy of analysis. Secondly, we assumed that all strokes were due to thrombus migration from the LAA to the central nervous system, however, other causes of stroke could not be excluded. Finally, the study included patients who already suffered from stroke and all studied parameters were measured after the stroke. Thus, we may only examine an associated between these parameters and history of stroke whereas their true predictive value should be confirmed in larger prospective study including patients who are at risk of stroke but have not yet developed this complication.

In conclusion, LASP is an independent risk factor for TE in patients with AF. The patients with higher LASP had a significantly lower incidence of previous stroke compared to those with lower values. The proposed new formula for identification of AF patients who are at risk of stroke, based on six geometrical measurements of LA and the sphericity coefficient and volume coefficient, is superior to the LASP in identification of AF patients with a history of ischemic stroke.

## Supplementary Information

Below is the link to the electronic supplementary material.

Fig. 1S Scatter plot of points belonging to three classes in the plane of canonical variables. Discrimination based on the left atrium geometry of 3 groups (1 – control group, 2 – stroke group without anticoagulant treatment, 3 – stroke group proper anticoagulation therapy). LD1 – first canonical variable, LD2 – second canonical variable (DOC 47 kb)
