# Supplementary figures and images for: Association of Left Atrial Sphericity with Risk of Stroke in Patients with Atrial Fibrillation. Sub-Analysis of the ASSAM Study
Source: Cardiovasc Eng Technol. 2021 Nov 8;13(3):419–27. doi: 10.1007/s13239-021-00587-y (PMC9197816; doi:10.1007/s13239-021-00587-y)

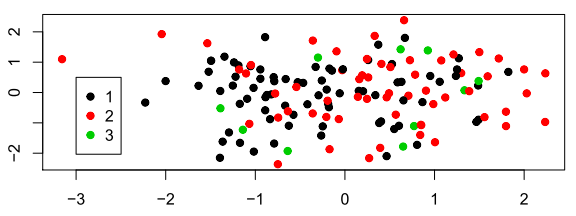


LD2

LD1

Supplement: Supplementary file 1 — Fig. 1S Scatter plot of points belonging to three classes in the plane of canonical variables. Discrimination based on the left atrium geometry of 3 groups (1 – control group, 2 – stroke group without anticoagulant treatment, 3 – stroke group proper anticoagulation therapy). LD1 – first canonical variable, LD2 – second canonical variable (DOC 47 kb) [file 13239_2021_587_MOESM1_ESM.doc]
